# New and personalized ventilatory strategies in patients with COVID-19

**DOI:** 10.3389/fmed.2023.1194773

**Published:** 2023-06-02

**Authors:** Lucas Rodrigues de Moraes, Chiara Robba, Denise Battaglini, Paolo Pelosi, Patricia R. M. Rocco, Pedro Leme Silva

**Affiliations:** ^1^Laboratory of Pulmonary Investigation, Institute of Biophysics Carlos Chagas Filho, Federal University of Rio de Janeiro, Rio de Janeiro, Brazil; ^2^Unit of Anaesthesia and Intensive Care, San Martino Hospital (IRCCS), Genoa, Italy

**Keywords:** COVID-19, noninvasive respiratory support, invasive mechanical ventilation, prone position, recruitment maneuvers, extracorporeal membrane oxygenation

## Abstract

Coronavirus disease (COVID-19) is caused by the severe acute respiratory syndrome-coronavirus-2 (SARS-CoV-2) virus and may lead to severe respiratory failure and the need for mechanical ventilation (MV). At hospital admission, patients can present with severe hypoxemia and dyspnea requiring increasingly aggressive MV strategies according to the clinical severity: noninvasive respiratory support (NRS), MV, and the use of rescue strategies such as extracorporeal membrane oxygenation (ECMO). Among NRS strategies, new tools have been adopted for critically ill patients, with advantages and disadvantages that need to be further elucidated. Advances in the field of lung imaging have allowed better understanding of the disease, not only the pathophysiology of COVID-19 but also the consequences of ventilatory strategies. In cases of refractory hypoxemia, the use of ECMO has been advocated and knowledge on handling and how to personalize strategies have increased during the pandemic. The aims of the present review are to: (1) discuss the evidence on different devices and strategies under NRS; (2) discuss new and personalized management under MV based on the pathophysiology of COVID-19; and (3) contextualize the use of rescue strategies such as ECMO in critically ill patients with COVID-19.

## 1. Introduction

Coronavirus disease 2019 (COVID-19) is caused by the severe acute respiratory syndrome-coronavirus-2 (SARS-CoV-2) virus and can lead to respiratory failure (1, 2). COVID-19 may manifest with different degrees of respiratory failure, up to acute respiratory distress syndrome (ARDS), named “C-ARDS” (3). Initially interpreted as viral pneumonia, its radiologic picture includes ground-glass opacities (GGOs), with large alveolar edema and consequent collapse and increase in blood volume and interstitial space. GGOs also have dilated vessels (4), with a risk of microthrombosis and endotheliitis (5). At hospital admission, patients can present with severe hypoxemia even under conventional oxygen therapy (COT) and dyspnea, which may require ventilatory support. The disease can develop heterogeneity among patients, and COVID-19 can assume different phenotypes (6). Three phenotypes have been described: L-type, characterized by low lung elastance; H-type, characterized by high lung elastance (7–9); and F-type, the final evolution of COVID-19 characterized by lung fibrosis (10–13).

However, depending on the severity of the disease, the need for supportive strategies may evolve to mechanical ventilation (MV) with the use of low tidal volumes (V_T_) (14, 15). If hypoxemia persists, prone position (PP) and alveolar recruitment maneuvers (ARM) can be considered (9, 12, 16–19). In addition, extracorporeal membrane oxygenation (ECMO) should be considered in the most severe cases of C-ARDS (17, 20–22). Recently, the literature has focused on individualization of ventilatory strategies, according to a broad range of patient variables (9, 10), including physiological data, lung imaging, laboratory data, biomarkers, and even omics data (10). However, some of these tools are not routine practice in many hospitals. Nevertheless, adopting personalized medicine could better implement the therapy in the patients with C-ARDS.

**Figure 1:**
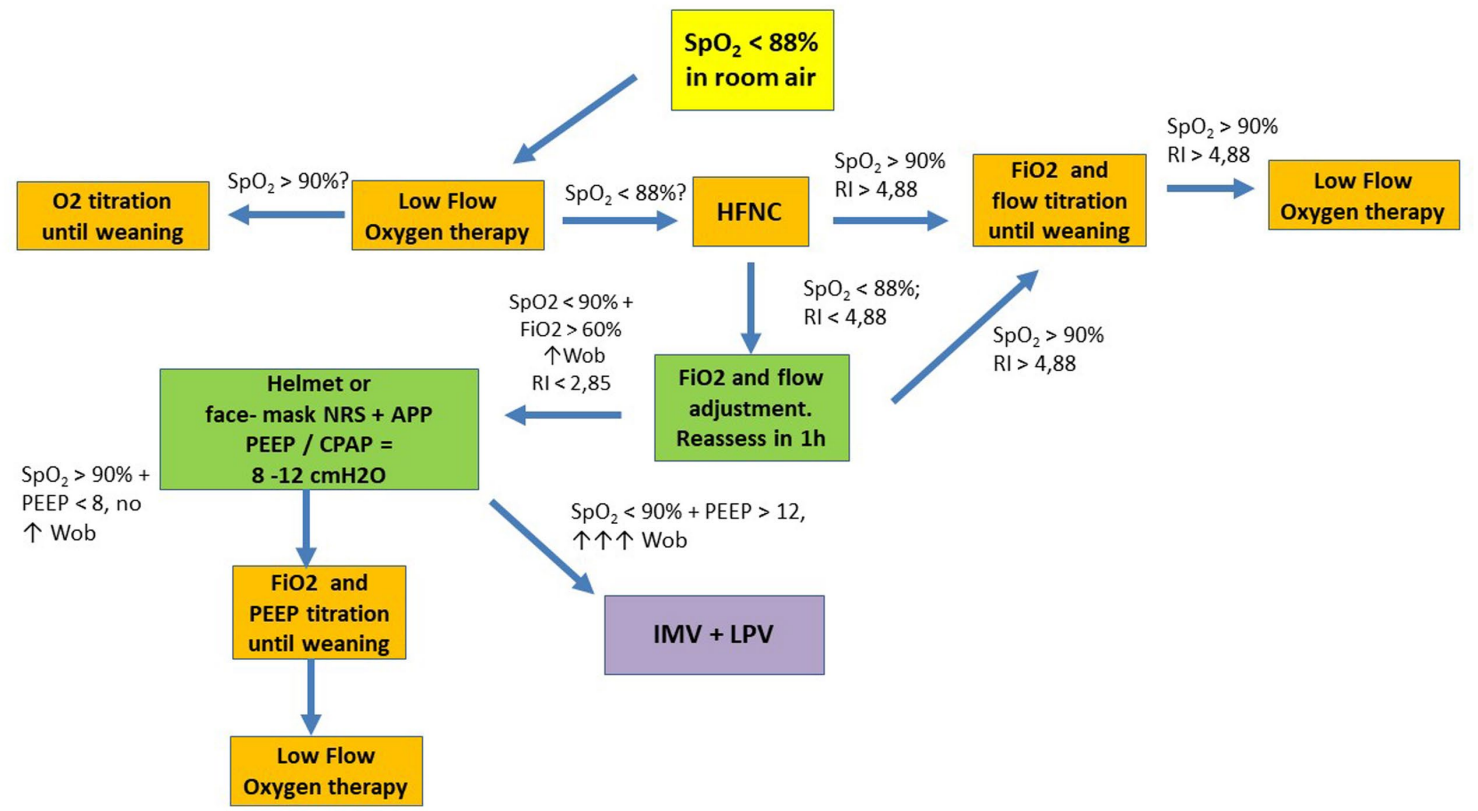
Suggested flowchart for noninvasive ventilatory management in COVID-19. APP, awake prone position; HFNC, high flow nasal cannula; LPV, lung protective ventilation; NRS, noninvasive respiratory support; P/F, PaO_2_ to FiO_2_ ratio; RI, ROX Index; SpO_2_, oxygen saturation; WOB, work of breathing; CPAP, continuous positive airway pressure; IMV, invasive mechanical ventilation; PEEP, positive end expiratory pressure.

The aim of this narrative review are to: (1) discuss the evidence on different devices and strategies for noninvasive respiratory support (NRS); (2) discuss new and personalized management under MV based on the pathophysiology of COVID-19; and (3) contextualize the use of rescue strategies such as ECMO in critically ill patients with COVID-19 (Figure 1).

## 2. Physiopathology and phenotypes

In the early stages of COVID-19, the virus targets nasal, bronchial, and pneumocytic epithelial cells. The spike protein of the virus binds to the angiotensin-converting enzyme 2 (ACE2) receptor (23), which allows the virus to enter the host cells, mainly into type II pneumocytes, where the virus starts to replicate. Subsequently, damage to endothelial cells occurs with consequent damage to the alveolar-capillary barrier, resulting in increased cell permeability (23).

The late phase is characterized by a large inflammatory cascade mediated by neutrophils and monocytes, which leads to large diffuse alveolar lesions (4, 5). In this phase, vascular lysis is often observed, with extensive destruction of the lung parenchyma and pneumocytes, alveolar collapse, and the formation of hyaline tissue (5). At the vascular level, dysregulation with stasis, microthrombi, microhemorrhages, and pulmonary embolism are commonly observed due to the high vascular permeability. The alveolar-capillary destruction caused by vascular lysis results in progressive hypoxemia and hypercapnia (4, 9, 24). At first, hyperventilation is noted. However, with progression of the inflammatory cascade, arterial partial pressure of carbon dioxide (PaCO_2_) levels increase and pH becomes acidic (5).

**Figure 2:**
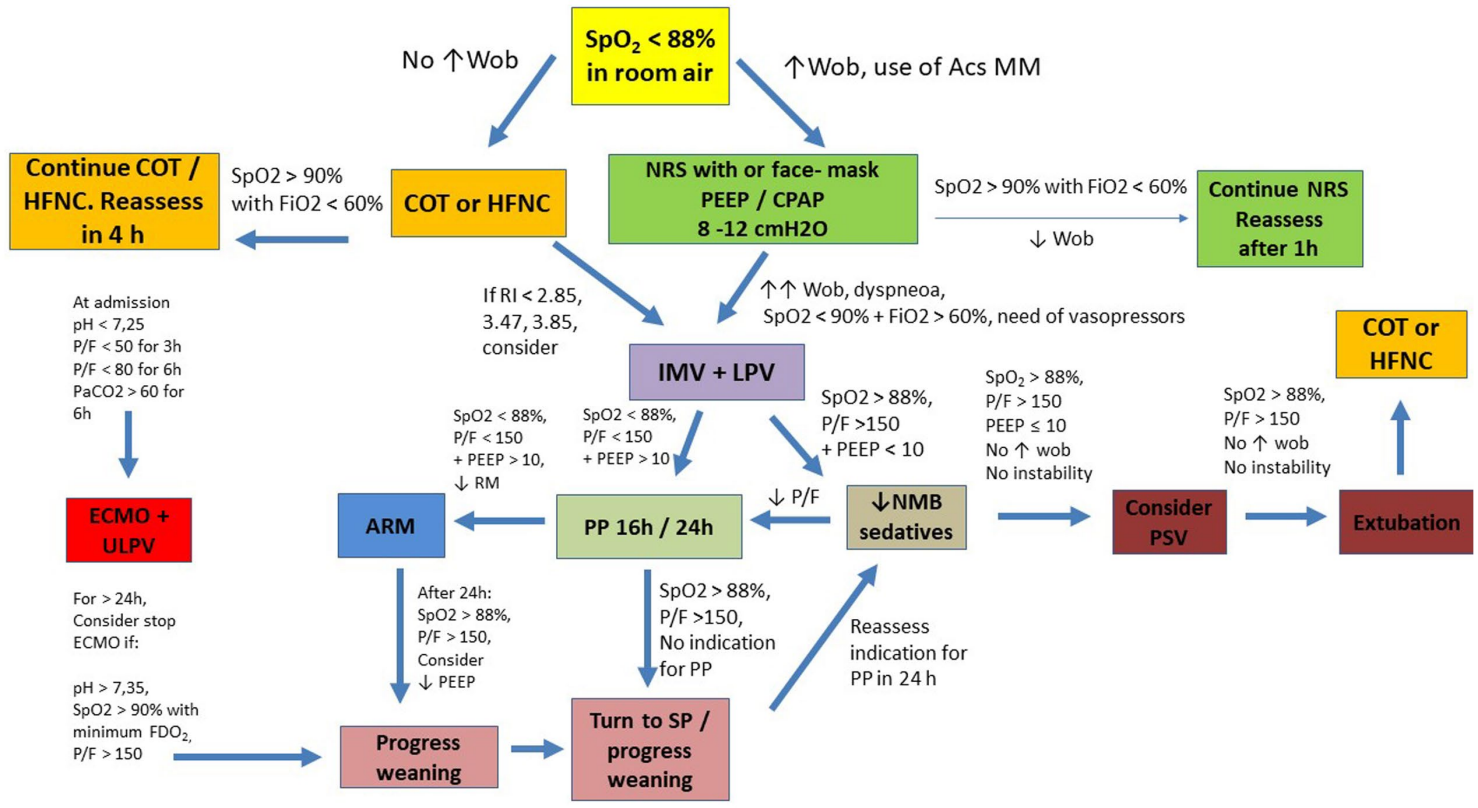
Suggested flowchart for ventilatory management in COVID-19. Acs MM: Accessories Muscles; ARM, alveolar recruitment maneuver; COT, conventional oxygen therapy; ECMO, Extracorporeal Membrane Oxygenation; IMV, invasive mechanical ventilation; HFNC, high flow nasal cannula; LPV, lung protective ventilation; NMB, neuromuscular blocker; NRS, noninvasive respiratory support; P/F, PaO_2_ to FiO_2_ ratio; PP, prone position; PSV, pressure support ventilation; RI, ROX Index; SP, supine position; SpO_2_, oxygen saturation; ULPV, ultra lung protective ventilation; WOB, work of breathing; CPAP, continuous positive airway pressure; PaO_2_, arterial partial pressure of oxygen; FiO_2_, fraction of inspired oxygen; FDO_2_, fraction of oxygen in the sweep gas stream; PEEP, positive end-expiratory pressure.

Faced with great alveolar damage, COVID-19 presents as a disease with severe hypoxemia (4, 5, 9, 24, 25). The development of the disease is characterized by the predominance of non-aerated lung tissue, mainly in the dependent regions of the lung. Under normal conditions, these regions have normal blood flow. However, in COVID-19, perfusion is observed to be antigravity, diverting to non-dependent (normally aerated) lung regions (8, 26, 27), with loss of the hypoxic vasoconstriction reflex (4, 6, 8, 27). One hypothesis is that there is a loss of response of sensitive chemoreceptors to low arterial partial pressure of oxygen (PaO_2_). Another possibility is dysregulation of mitochondria and the pathways involved in oxygen sensing (26). Ventilation/perfusion (V/Q) dysregulation is observed, which is initially due to the presence of hyperperfused ground-glass regions (8, 9, 24, 26, 27). In later stages, the formation of atelectasis is observed, distributed non-homogeneously. V/Q irregularity remains due to the presence of extremely non-aerated areas (8, 9, 24, 26, 28). In autopsy studies, lungs with confirmed SARS-CoV-2 infection exhibit paste within the alveolar cavity, fibrinous exudation, proliferation of type II alveolar epithelial cells and macrophages, vascular congestion of the alveolar septum, and vascular thrombi (23). This points to the importance of the vascular bed in the development of COVID-19 pneumonia (Figure 2).

Some authors divide the histopathology of COVID-19 into three phases that resemble pulmonary ARDS due to the presence of diffuse alveolar damage; (1) the acute/early phase, characterized by intra-alveolar edema and interstitial widening, with peak hyaline membrane formation, both diffuse and focal, which occurs between 4 and 5 days after the initial insult; (2) the organizer stage, also called myeloproliferative, characterized by intense cell proliferation of fibroblasts and hyperplasia of type II epithelial cells; (3) the late/fibrous stage with honeycombing (8, 23, 26).

Three COVID-19 phenotypes can be established to broaden understanding of the pathophysiology (4, 8, 9, 24–27), although the literature does not recommend this (9).

### 2.1. COVID-19 phenotypes

#### 2.1.1. The L phenotype

The L phenotype occurs in mild to moderate cases, mainly in the early stages. It is classified as low respiratory system elastance, low ventilation-to-perfusion ratio, low lung weight, low lung recruitability (25). It is the longest surviving phenotype (25). It is characterized by hyperperfused subpleural focal GGOs maintaining lung areas that are normally aerated (4, 8, 9, 24–27). Increased perfusion can lead to capillary collapse and hypercoagulability/microthrombosis, leading to deviation of blood flow to the non-dependent regions of the lung (very aerated) and resulting in loss of the hypoxic vasoconstriction reflex (4). Thus, an increase in poorly perfused dependent areas is observed. This situation decreases the V/Q ratio. The clinical picture includes severe hypoxemia with satisfactory ventilatory mechanics (“happy hypoxemia”) (4, 5). Low lung weight is observed, and compliance of the respiratory system is normal or minimally reduced. Therefore, the percentage of poorly aerated tissue is low, as is recruitability. Zubieta-Calleja et al. (23) suggest that this happy hypoxemia may be due to a reduced ventilatory drive, commonly found in this phenotype. Given the dissociation between the extent of hypoxemia and normal compliance, two explanations have been proposed to characterize the severe hypoxemia. The first is the focality of the lung lesion, as demonstrated by the ground-glass pattern. This partially reduces ventilation without affecting elastic recoil. Because there is great lung perfusion, low V/Q areas are diffusely distributed throughout the lung from ventral to dorsal and cranial to caudal (4, 5, 8, 9, 24–27). Pulmonary involvement is low at this stage, therefore the patient’s ventilatory work is still normal. A second explanation is that gas exchange abnormalities arise primarily from vascularly mediated injury, which is not observed at this stage (25). Gattinoni et al. (6) suggest that hypoxemia is due to perfusion irregularity and that vasoplegia is also responsible for low PaO_2_. In addition to diverting blood flow, ventilation is directed toward non-dependent regions, which allows the creation of dead space areas (5). In conjunction with low V/Q lung units, this phenotype is considered to have wasted ventilation, which does not substantially affect oxygenation (8). Patients with this phenotype may be candidates for NRS or high flow nasal oxygen to correct hypoxemia (8, 29). This phenotype can also be found in promptly intubated patients.

As the disease progresses, the L phenotype may progress to the H phenotype, characterized by low lung compliance. One of the signs of transition between phenotypes is the need for a high fraction of inspired oxygen (FiO_2_) and increased ventilatory drive (8, 25). Increases in inspiratory efforts are directly associated with worsening of inflammation and, in turn, with increased V_T_ and increased vascular permeability with the formation of alveolar edema. This is one of the mechanisms of patient self-inflicted lung injury (P-SILI) (discussed in Section “Patient self-inflicted lung injury in NRS”). In addition, NRS should be considered with utmost caution in patients with L phenotype, because progression from type L to type H phenotype can be also caused by mechanisms of inflammatory amplification overlapping the host inflammatory response phase (25). Over time, alveolar edema increases and lung volume decreases, reducing the lung area available for gas exchange (8, 9, 24–27). Thus, the inspiratory volumes generated for a given inspiratory pressure decrease, resulting in dyspnea. At this time, transition from the L to the H phenotype is expected (8, 25).

#### 2.1.2. The H phenotype

The H phenotype represents evolution of the L phenotype and is found in critically ill patients (8, 9, 24–27). In this phase, there is amplification of the inflammatory response, allowing greater cellular permeability and formation of alveolar edema (25). As a result of the decrease in gas volume during the evolution of the L phenotype, an increase in lung weight is observed due to the presence of irregularly distributed consolidated areas, predominantly in the dependent regions. This leads to an increase in lung elastance and, in turn, a decrease in lung compliance, with the development of a restrictive pattern of ventilation. Alveolar units of low V/Q ratio are increased (8, 9, 24–27). In this phenotype, however, this happens due to increased lung consolidation, unlike the L phenotype, where the explanation rested on GGOs (8). By increasing pulmonary edema and the pressure exerted on the lung parenchyma, the cardiac output perfusing non-aerated lung areas contributes to the formation of a right-to-left shunt (6). As in the L phenotype, wasted ventilation persists to a great extent (8). The H phenotype has a high capacity for alveolar recruitment (Section “Alveolar recruitment maneuvers and PEEP titration”).

#### 2.1.3. The F phenotype

Faced with the ventilatory dysfunctions found in the two previous phenotypes, Tonelli et al. (25) considered the final pathway of COVID-19 to be the development of pulmonary fibrosis, namely the F phenotype (8, 25, 30).

The evolution of the L to H phenotype is mediated by an intense inflammatory cascade (24). During its evolution, there is activation of multiple aberrant inflammatory pathways that unbalance the relationship between pro-fibrotic and anti-fibrotic mediators (25). In the F phenotype, fibroproliferation occurs so that the lung resembles a patchwork quilt. This causes the alveolar units to have different lung elasticities, with different capacities for volumetric accommodation (8, 9, 24–27).

During spontaneous ventilation, some alveolar units may be more distensible than others, generating high transpulmonary pressures with a high risk of lung injury and pneumocyte rupture (8, 9, 24–27). Furthermore, the pulmonary fibrotic pattern found in this phenotype reduces carbon dioxide diffusing capacity, leading to hypercapnia (8, 25). Rescue therapies such as alveolar recruitment and PP are not very effective because there is a high density of collagen, which is not easily distensible.

## 3. Noninvasive respiratory support

At hospital admission, patients with COVID-19 may present with low PaO_2_ and dyspnea. Both can be explained by silent hypoxemia and the presence of non-ventilated areas, as shown by computed tomography, with important ventilation-perfusion inequalities (2, 31). NRS is able to correct hypoxemia, reduce the work of breathing, and improve poor ventilated areas (28), and, even in some scenarios, endotracheal intubation can be avoided (31).

### 3.1. The choice of NRS interface

Overall, two strategies have been adopted: continuous positive airway pressure (CPAP) and BILEVEL, i.e., pressure support (PS) +: positive end expiratory pressure (PEEP) with the use of two interfaces: facemask and helmet (1, 2, 15, 28, 31, 32). Both interfaces improve oxygenation and reduce aerosolization compared with a high flow nasal cannula (HFNC) and COT.

Patients with COVID-19 may require prolonged NRS therapies (due to low oxygenation on admission) to avoid MV. In this case, there might be a need to apply high PS of up to 12 cmH_2_O (15, 29, 32), PEEP values between 8 and 12 cmH_2_O (2, 15, 29, 32), reaching up to 15 cmH_2_O (33) and as little air leakage as possible. The helmet is one of the best interfaces to promote patient comfort (1, 2, 15, 34). It is also associated with reduced intubation rates and better correction of hypoxemia (35). However, the helmet is associated with greater rebreathing of CO_2_, requiring intensive monitoring (36). Furthermore, a randomized clinical trial by Arabi et al. (34) divided 320 patients into two groups: half used a helmet and the other half received COT. No differences in mortality were observed after 28 days, but helmet mortality was observed at day 180. Compared with COT, no differences in mortality were observed (37).

### 3.2. Parameter adjustments during NRS

The best comfort level can be achieved by adjusting ventilation parameters, such as PS and PEEP levels. The PS level is associated with the generation of V_T_ necessary to ensure adequate aeration; the PEEP level is responsible for ensuring oxygenation. A retrospective study reported the use of CPAP in 46 patients with PEEP ranging between 8 and 12 cmH_2_O. The PEEP level was adjusted according to clinical tolerance, air leakage, and peripheral saturation of oxygen (SpO_2_) (31). Only nine patients were intubated between days 7 and 14, and the authors recommended the use of CPAP to avoid intubation. Similar results were found in another study that compared HFNC with CPAP in 151 patients (98% with helmet) and NRS in 72 patients (15 with a helmet and 57 with a facemask) (1). One hundred sixty-three patients received HFNC. For the first two interfaces, the authors established a mean PEEP level of 10.2 cmH_2_O during CPAP and 9.5 cmH_2_O in NRS. Although all the interfaces were shown to improve oxygenation, there was no difference in intubation rates and length of stay (1). In an important randomized clinical trial, COT (low or high flow) was compared with CPAP adjusted to a mean PEEP of 8.2 cmH_2_O. Only 36% of the patients in the CPAP group were intubated compared with 44% of the HFNC group. In addition, CPAP reduced mortality compared with COT (26). More recently, Colaianni-Alfonso et al. (35) studied 112 patients with moderate to severe COVID-19 who failed HFNC; the patients were divided into two groups for CPAP: one group used a facemask interface with median PEEP of 12 cmH_2_O and the other a helmet interface with median PEEP of 14 cmH_2_O. The groups remained on continuous CPAP for 24 h. It was observed that the helmet group had lower intubation rates and a more marked improvement in oxygenation compared with the facemask group, which had higher intubation rates and longer length of stay. Although these data favor the helmet, caution is required in the interpretation, because the PEEP value applied must be consistent with the clinical condition of the patient.

Adjustments other than PS and PEEP can be fine-tuned at the bedside. In 2009, a study (36) evaluated 13 patients after extubation and randomly performed three 20-min periods of NRS with three interfaces: facemask, helmet, and helmet with a 50% increase in PS and PEEP associated with a high rate of pressurization (rise time). Using the first two interfaces, PS had a mean level of 10 cmH_2_O, PEEP of 5 cmH_2_O and 0.2 s of pressurization time. In the third group, PS had a mean level of 15 cmH_2_O, PEEP of 8 cmH_2_O, and the shortest possible pressurization time, i.e., 0.05 s. The authors analyzed transdiaphragmatic pressure (Pdi), which is a surrogate of inspiratory effort through an esophageal catheter. Pdi was reduced in the helmet group with higher PS (15 cmH_2_O) and PEEP (8 cmH_2_O) and fast pressurization time (0.05 s). This highlights an important comparison between the facemask and helmet interfaces. Keeping the same cycling-off (25%), the helmet had more asynchrony events at the end of inspiration compared with the facemask, with ventilator cycling sooner or later compared with the end of the patient’s inspiratory time. In this case, the ventilator’s inspiratory time was shorter than the patient’s neural time. The authors point out that an overlap exists between the PS applied by the ventilator and the patient’s neural time. As an explanation, the authors hypothesize that cycling with the helmet seems to occur due to changes in flow caused by the mechanical characteristics of the interface and not by the characteristics of the patient. With the patient’s inspiratory time longer than that of the helmet, the interface promotes minimal reduction in ventilatory overload. Further studies on patients with COVID-19 comparing the two interfaces are necessary for a better understanding of their ventilatory repercussions.

The choice of the ideal interface, as well as fine ventilator adjustments, should aim to achieve patient-ventilator synchrony, reduce the work of breathing, especially with the helmet interface and to ensure comfort. A multicenter randomized clinical trial randomized 54 patients (mean age, 66 years) to NRS and 55 to HFNC (15). The NRS group underwent therapy for at least 48 h and used a helmet interface with PS and PEEP levels ranging between 10 and 12 cmH_2_O, no pressurization time (rise time), expiratory trigger between 10 and 50% to avoid double trigger, inspiratory trigger to avoid auto-triggering, and maximum inspiration time between 1 and 1.2 s. PS was titrated individually to ensure high flows to the patient. The NRS group showed lower intubation rates and more MV-free days compared with the HFNC group. This allows us to conclude that the success of NRS is based on fine ventilatory adjustments.

Typically, NIV is a therapy performed in the ICU. However, with the pandemic exceeding the capacity of available beds, this tool gained space outside the ICU. Cammarota et al. (37) conducted a systematic review with meta-analysis including 17 articles containing 3,377 patients which showed the effectiveness of NIV outside the ICU environment as an adequate tool to deal with the demand for ventilatory assistance.

### 3.3. NRS therapeutic targets

The literature is not concordant regarding the therapeutic objectives of NRS. Perkins et al. (28) indicated different factors as therapeutic targets, such as: SpO_2_ > 90%, respiratory rate ≤ 25 bpm, and a reduction in the work of breathing (26). Aliberti et al. (2) stated that NRS weaning can be performed if the patient’s SpO_2_ > 94% with FiO_2_ < 50% and PEEP ≤5 cmH_2_O. Arabi et al. (34) stated that application of PEEP should target SpO_2_ between 92 and 98%, and that the respiratory rate should be <25 bpm. More recently, Colaianni-Alfonso et al. (35) state that application of PEEP should target an SpO_2_ between 92 and 96%.

### 3.4. Predictors of NRS failure

After starting NRS, patient monitoring must be constant to assess its effectiveness or failure. Arabi et al. (34) suggest assessment every 1 to 3 h, but this may vary according to the intensive care unit (ICU). In case of therapy refractoriness, the literature indicates that MV should not be postponed. Some signs of failure mentioned in the literature are: respiratory rate > 40 bpm, respiratory acidosis with pH <7.25–7.30, use of accessory muscles, dyspnea, swallowing disturbance, SpO_2_ < 88–90% for more than 5 min, PaO_2_/FiO_2_ ratio < 100, persistent requirement for FiO_2_ > 70%, hemodynamic instability (systolic blood pressure < 90 mmHg or mean blood pressure < 65 mmHg, even with volume resuscitation), deterioration in the level of consciousness (2, 15, 34, 35). Contrary to the data, the intubation criteria in the randomized clinical study by Perkins et al. (28) are stricter. These authors compared CPAP with low and high flow oxygen therapy and considered an SpO_2_ ≤ 94% with an FiO_2_ of at least 40% to be a ventilatory risk. Robba et al. (27) suggest immediate intubation if the PaO_2_/FiO_2_ ratio does not improve and/or PaCO_2_ < 30 mmHg and/or respiratory rate > 28 bpm using accessory muscles for more than 3 h. More recently, the study by Colaianni-Alfonso et al. (35), who compared helmet CPAP and helmet facemask (discussed earlier), considered pH <7.35 as a criterion for intubation, in addition to all previous signs. These data indicate that there is no clear guideline for the signs of NRS failure, allowing the use of some scales to help diagnose it (Figure 1).

### 3.5. NRS failure prediction scales

The literature presents some scales/indices that help in the diagnosis of therapeutic failure during NRS:

- ROX Index (38–40)
- Sepsis-related Organ Failure Assessment (SOFA) (41)
- HACOR Score (heart rate, acidosis, consciousness, oxygenation, and respiratory rate) (42–45)
- Simplified Acute Physiology Score (SAPS) (46)

#### 3.5.1. ROX index

Originally developed to assess the effectiveness of HFNC, the ROX Index has been used as a predictor of the success or failure of NRS. It consists of dividing the SpO_2_/FiO_2_ quotient by the respiratory rate. It must be calculated in periods of time not yet defined in the literature, but which may be the same as the HFNC. Values < 2.85, <3.47, and < 3.85 after 2, 6, and 12 h, respectively, have been demonstrated to be predictors of therapy failure (38). At the same cutoff points, values ≥ 4.88 indicate success of NRS (38). A recent article applied CPAP with a mean PEEP of 12 cmH_2_O in 112 patients with a facemask interface. All patients remained on CPAP for 24 h (39). The researchers calculated the ROX Index after 2, 6, 12, and 24 h of positive airway pressure, and values <6.64 after 24 h of therapy were associated with therapeutic failure. The cutoff periods of 2, 6, and 12 h showed low specificity and sensitivity (39). Higher cutoff points were found in an American study in 2022 (40). The researchers applied CPAP in 95 patients with an initial PEEP of 5 cmH_2_O. FiO_2_ was adjusted individually by SpO_2_. The ROX Index was measured after 2, 6, 12, 18, and 24 h of positive airway pressure, and values < 8.76, <9.08, <9.50, <8.58, and < 7.77, respectively, were predictors of NRS failure. However, details of the interfaces were not given (34).

#### 3.5.2. Sepsis-related organ failure assessment

SOFA was developed in 1996 (41) to assess multiorgan failure, which is a characteristic of COVID-19 (30). It includes six domains each with scores between 1 and 4: breathing, coagulation, liver, cardiovascular, neurologic, and renal. Values > 2 indicate the presence of sepsis and, therefore, a risk of mortality. A recent prospective study (47) evaluated 1,491 patients, 158 of whom received NRS; the rest received low flow or high flow oxygen therapy. Mean PS was 8 cmH_2_O, mean PEEP was 7 cmH_2_O, and mean FiO_2_ was 60%. Patients on NRS had a mean SOFA score of 3. This group had higher intubation and mortality rates at 28, 60, and 90 days. Although the authors did not provide a cutoff point for NRS failure, they showed that most patients had a score > 3 in the cardiovascular domain after 24 h in the ICU. Previously, another prospective study (48) included 58 patients received NRS. Twenty-seven patients who progressed to intubation had an average of 4 points on the SOFA; the group who were not intubated had 3 points. Furthermore, the authors showed that high scores on the scale were associated with low oxygenation. Although the authors did not explain this association, within the respiratory domain of SOFA, a score of 4 indicates a PaO_2_/FiO_2_ ratio < 100, suggesting therapeutic failure. They concluded that high SOFA was related to intubation but did not provide data on mortality. These data are in agreement with previous studies (1). With the facemask and helmet interfaces, the average SOFA values were 3.3 and 4, respectively. The difference in scores between the interfaces remains unknown.

#### 3.5.3. HACOR score

Originally developed in 2017 (42), the HACOR Score consists of five parameters easily collected at the bedside: heart rate, acidosis, consciousness, oxygenation and respiratory rate. Of these domains, four are included in the evaluation of the effectiveness of NRS, which makes this score very accurate in detecting therapeutic failure (43). The authors reported that the cutoff point for NRS failure must be ≥5. The study by Innocenti et al. (44) evaluated 135 patients who underwent CPAP with a full-face or oronasal mask. The HACOR Score, ROX Index, and SOFA scales were applied 3 days and 1 day before the start of NRS, on the day of admission, and on days 1, 2, 5, 8, and 11 after NRS. The authors did not provide information about PEEP adjustments. FiO_2_ was titrated to achieve an SpO_2_ of 94%. Thirty-five patients died (considered as a therapeutic failure) given the presence of several comorbidities. This group had a HACOR Score > 5, ROX Index <4.88, and SOFA scores ≥4.

An observational study by Guia et al. (43) evaluated the HACOR Score of 128 patients with a mean age of 61 years after performing 1 h of CPAP with a mean PEEP of 10 cmH_2_O. Thirty-two patients had a HACOR Score ≥ 5, and 22 failed therapy; i.e., 69% of the positive predictive value. On the other hand, 96 patients had a HACOR Score < 5 points. Of these, 83 had successful CPAP; i.e., 86% of the negative predictive value.

#### 3.5.4. Simplified acute physiology score

Originally developed in 1993 by Le Gall et al. (46), SAPS includes cardiorespiratory and renal parameters, laboratory analysis of red and white blood series, and electrolytes. It is a larger scale than the previous ones. Values vary between 0 and 163 points. Higher scores are associated with worse prognosis. According to the original study, a SAPS of 29 points is associated with 10% of deaths, and values of 40 are correlated with 25% of deaths. Very few studies on COVID-19 included in this review used SAPS. Patients in the study by Oranger et al. (31) showed SAPS of 26 points, whereas the study by Grieco et al. (15) reported mean values of 32 points. The multicenter study by Schmidt et al. (47) reported that patients with COVID-19 on NRS had a mean SAPS of 33 points. It is reasonable to conclude that there is a correlation between SOFA, SAPS II, HACOR Score, and the ROX Index regarding the diagnosis of NRS failure.

### 3.6. Patient self-inflicted lung injury in NRS

At the beginning of the pandemic, the initial recommendation was early intubation to protect the lungs (3, 49). Due to the urgency caused by the pandemic, limited staff, and few noninvasive ventilatory resources to meet the demand, many patients experienced worsening respiration and consequent intubation.

With the reduction in the number of cases, noninvasive ventilatory support such as NRS and HFNC was introduced with the aim of reducing ventilatory effort and avoiding intubation (29). However, when instituting noninvasive therapy, adequate monitoring is required to avoid P-SILI (8, 27, 50). In patients with COVID-19, reduced lung compliance and heterogeneous distribution of inspired V_T_ are observed due to the presence of areas of low V/Q ratio that are distributed irregularly throughout the lung (4, 8, 24, 25, 51). In spontaneous ventilation, during the inspiratory phase, sufficient diaphragmatic contraction is required to counteract pulmonary elastic recoil forces (8, 24, 25, 27, 50). This generates large variations in transpulmonary pressure (P_L_).

In the early stages of COVID-19 (L phenotype), when there are no large pulmonary consolidations, no variations in transpulmonary pressure are observed, which allows the application of NRS with greater safety (6, 25, 29). Under normal conditions of spontaneous breathing, during the inspiratory phase, pleural pressure decreases uniformly, whereas PL increases uniformly (50). In situations of increased ventilatory drive, greater inspiratory efforts are observed to generate a given V_T_ caused by greater negative pleural pressure, increasing P_L_, which reflects inspiratory efforts. This allows non-homogeneous distribution of lung pressures and volumes, leading to P-SILI (8, 25, 50). Expiratory efforts can also cause P-SILI (50). During intense expiratory activity, pleural pressure increases, drastically reducing P_L_, with alveolar collapse occurring in most dependent lung regions and peripheral airways. Battaglini et al. (50) suggest a study of stress/strain for a better understanding of the disease. Stress is the distribution of force applied per unit of lung area, and strain evaluates the stretching of this alveolar unit and is directly proportional to stress (25, 50). During the development of COVID-19, ventilatory efforts become more vigorous, leading to regional hyperdistention, especially in non-dependent regions, and further compromising dependent regions (8, 24, 25, 50). Therefore, both stress and strain are increased. This is associated with the development of pneumothorax and pneumomediastinum (50).

#### 3.6.1. Pendelluft phenomenon

Inspiratory pendelluft is a phenomenon found in the development of COVID-19 and contributes to the genesis of P-SILI (8, 25, 50, 52). It is defined as the disorganized distribution of gas when the inspiratory effort has not yet produced an inspiratory flow at the airway opening. It occurs due to different regional time constants or negative fluctuations in pleural pressure in patients who are breathing spontaneously. This allows irregular distribution of V_T_ and, consequently, of P_L_ (8, 25). In the pendelluft phenomenon, the gas moves from the non-dependent region to the dependent region, which remains under significant recruitment and hyperdistention and may release inflammatory mediators. Analyzing the transition of phenotypes is also useful to monitor P-SILI.

In the L phenotype, when lung compliance is normal or slightly reduced, a fluid-like behavior is predominant. Thus, the distribution of pleural pressure is homogeneous along the lung surface (25). With the worsening of inflammation and alveolar edema, this pulmonary phenotype can progress to type H (25). One of the signs of phenotypic transition is an increased respiratory rate (even in NRS), resulting in intense respiratory efforts (8, 25). Another sign of phenotypic transition is an increase in PEEP and an increase in FiO_2_ to maintain SpO_2_ > 90% (50). The generation of high V_T_ values can also indicate a phenotypic transition. When positive airway pressure is applied, P_L_ may increase with consequent production of high V_T_ outside the protective concept, i.e., between 6 and 8 mL/kg of predicted body weight (PBW) (33, 47, 50). This increases the chances of barotrauma.

Considering the pendelluft effect, the chances of P-SILI also increase. The gold standard for detecting ventilatory effort is esophageal pressure through a catheter that rests just above the diaphragm. However, its use is still restricted to experimental studies, not yet viable at the bedside (50). Tonelli et al. (52) proposed that measuring the variation in nasal pressure (P_nos_) is directly related to the variation in esophageal pressure (P_es_). For this, they studied 61 patients, of which 83% tested positive for COVID-19. The authors calculated both pressures. They used a nose clip for the analysis of P_nos_ and asked the patients to keep their mouth closed throughout the evaluation. On the third day of NRS, the authors observed that patients who evolved to invasive MV had a mean ΔP_es_ of 14 cmH_2_O and a mean ΔP_nos_ of 6.5 cmH_2_O. The values for those who remained in NRS were 12 and 5.6 cmH_2_O, respectively. This was an early cohort study. New studies are important to confirm this information.

In addition, asynchrony events are also associated with the genesis of P-SILI; double triggering is the most common. In patients with COVID-19, the expiratory phase is marked by a significant increase in pleural pressure, reducing pleural pressure, causing collapse of most dependent lung regions and peripheral airways. Hence, P-SILI is also influenced by the pendelluft effect. This leads to alveoli with different regional time constants.

#### 3.6.2. Squishball phenomenon

During the transition from the H to F phenotype, a severe increase in esophageal pressure is observed as the lung is assuming a pattern of fibrosis or a patchwork. There is deposition of collagen and elastin, poorly contractile proteins, therefore the chance of P-SILI and ventilator-induced lung injury (VILI) increases dangerously if the patient remains on NRS (8, 25). During the inspiratory phase, fibrotic lungs present heterogeneous behavior, because lung tissue does not have the same mechanical properties in all directions when a given transpulmonary pressure is applied (8, 25). In addition, the application of PEEP or high V_T_ can determine hyperdistention of more distensible lung areas (34). This is called the squishball phenomenon, which increases regional stress and strain (25). Its understanding is similar to the pendelluft effect.

#### 3.6.3. Mechanical power for monitoring P-SILI

Considering that the amount of energy to which the lung is subjected, even during assisted spontaneous breathing, can be crucial in the development of P-SILI, application of inappropriate ventilator pressure or the phenotypic evolution of the disease increase the patient’s esophageal pressure, resulting in VILI. Mechanical power can be assessed at the bedside to evaluate this phenomenon in a simple way (8) using the formula 0.098 × respiratory rate × V_T_ × (P_peak_ − 0.5∆P_aw_), where P_peak_ is the peak pressure and P_aw_ is the airway pressure. This index may represent a reliable estimate of the amount of energy transferred from the respiratory muscles and ventilatory assistance to the lung during assisted spontaneous breathing (8). Thus, the need for ventilatory adjustments, such as increased PS or PEEP, can be assessed.

The use of mechanical power is useful to assess pulmonary recruitability at the bedside (8). Decreased dynamic compliance is correlated with increased mechanical power and may suggest limited lung recruitability and predict the risk of local overdistention (8).

#### 3.6.4. P-SILI and perfusion irregularities

Another factor that increases the chances of P-SILI is the irregularity of lung perfusion (4, 5, 24, 26, 50). With increased inspiratory effort, pulmonary capillaries can be compressed, increasing pulmonary resistance. This leads to increased transalveolar and transcapillary pressures recruiting previously collapsed capillaries (50). On the other hand, it leads to hyperdistention of those located in healthy areas and in ground-glass regions, which can lead to increased blood flow in injured regions and damage to the alveolar-capillary membrane (50). This predisposes the formation of interstitial and alveolar edema, increasing the risk of P-SILI (25). With this, the phenomenon of pendelblut is observed, in which traction forces applied to vessels adjacent to stress generators can generate a blood siphon effect toward areas of greater P_L_ (8).

All these factors may lead to higher lung perfusion and predispose the formation of interstitial and/or alveolar edema and worsening lung inflammation (47). This may explain why patients intubated at a late stage are not responsive to PEEP and have low static compliance, increasing mortality (51). Despite the signs of NRS failure mentioned earlier, and considering the heterogeneous development of the disease among patients, the decision to intubate needs to be taken after discussion with a multidisciplinary team (2).

#### 3.6.5. Early versus late intubation

The decision to intubate should be made considering the course of the disease and the patient’s clinical condition. It should be performed in cases of complete refractoriness to NRS. However, with the reduction in the number of cases, patients under NRS can be better monitored, allowing for a lower rate of intubation.

The L phenotype normally appears hypoxemic, with no change in compliance. In this case, HFNC and NRS are first-choice interventions, because the patient still benefits from the therapy (25) and orotracheal intubation can be postponed.

With evolution from the L to the H phenotype, consolidations and alveolar collapse, which need to be reopened to ensure adequate oxygenation and reduction of ventilatory work, are present (8, 25). The problem is that the patient must develop extra diaphragmatic force due to the increase in elastic recoil (25). NRS at this point starts to become contradictory because the patient increases inspired V_T_ to overcome the elastic recoil leading to P-SILI. Robba et al. (27) stated that patients who remain on NRS for a long time may develop the H phenotype, which may result in diaphragmatic dysfunction. At this point, orotracheal intubation is recommended.

It is difficult to ventilate patients who have the F phenotype because the lungs present great heterogeneity in gas distribution, leading to the pendelluft effect (8, 25, 27). Maintaining spontaneous ventilation in this phenotype may increase the release of inflammatory mediators, and therefore intubation is recommended (8, 25).

Prolonged endotracheal intubation is associated with a worse prognosis, the need for emergency airway management (27), and increased mortality (18, 38, 47). Wendel-Garcia et al. (42) showed that compromised respiratory system mechanics during prolonged endotracheal intubation may explain the increase in mortality observed under NRS. It may also make it difficult to maintain protective ventilation and contraindicate ARM or PP due to increased areas of pulmonary consolidation and/or a radiologic pattern similar to fibrosis.

In a study by Ball et al. (41), 52 patients with a mean age of 64 years who failed helmet CPAP after a minimum of 2 h were divided into two groups: early intubation and late intubation, with a cutoff point of 2 days. After endotracheal intubation, patients underwent computed tomography imaging with two levels of PEEP: 8 and 16 cmH_2_O to assess ARM. The late intubated group had lower static compliance and a lower P/F ratio. Regarding ventilation distribution, the late intubated group had a higher percentage of poorly and non-aerated areas. Furthermore, this group did not respond to increased PEEP (8 to 16 cmH_2_O), requiring higher FiO_2_, indicating that these patients were not recruitable. There was no difference in mortality between the groups.

There is still a lack of studies in the literature that quantify the results of patients intubated early or late after NRS failure. The research carried out for this paper allowed the creation of Table 1.

**Table 1:** Selected NRS studies.

**Trial**	**Population**		**Intervention**		**Outcome**	
(sample size)	Inclusion criteria	Exclusion criteria	Treatment	Control	Primary	Secondary
**1) Retrospective Studies (n = 4)**						
Franco et al. (1)(n = 670)	- SpO_2_ < 94%, - RR > 20 and Poor response to 10–15 L/min COT - Requiring CPAP / NRS with high FiO_2_ - P/F < 200 requiring IMV	- SpO_2_ > 94%, - RR < 20 without need of COT or SpO_2_ < 94%, RR > 20 but responds to 10–15 L/min COT	CPAP or NRS or HFNC Interface: Helmet or Face mask PP applied in patients with bilateral posterior infiltrates.	No control group	↑ Oxygenation ↔ Mortality at 30^th^ day; ↔ IMV %; ↔ Hospital LOS	No described
Aliberti et al. (2)(n = 157)	Pneumonia as the only cause of hARF; P/F ratio < 300 during COT	- Immediate IMV; - GCS <15; - Respiratory acidosis; - Need of Vasopressors; - Risk of aspiration pneumonia; - Inability to protect airways	CPAP using helmet interface. PEEP = 10.8±2.3 cmH2O	No control group	↑ IMV % ↑ Mortality in ICU	↑ CPAP success ↑ Mortality at 30^th^ day;
Oranger et al. (31)(n = 52)	COT > 6 L/min to SpO_2_ ⩾ 92%	No described	CPAP 8-12 cmH_2_O Interface: Face mask.	COT up to 15 litres/min.	↓ IMV % at 7^th^ and 14^th^day ↓ Mortality in DNI patients	No described
Wendel-Garcia et al. (33) (n = 1093)	- bilateral infiltrates in the chest X-ray - need COT to keep SpO_2_ ≥ 90%	- IMV before and after ICU admission - COT or combination with HFNC and NRS	NRS Group Interface: Not mentioned PS: The necessary to generate V_T_ of 5.7-7.6 ml / PBW PEEP: 12-15 cmH_2_O	COT: Litrage not mentioned HFNC: Flow: Not mentioned FiO_2_: 50-70%	↓ IMV in HFNC ↔ ICU LOS ↔ ICU Mortality ↓ VFD in COT	No mentioned
**2) Prospective Studies (n = 3)**						
Ranieri et al. (32) (n = 315)	- hARF - Bilateral opacities on chest X-ray - P/F ratio < 300 mmHg - Previous treatment for hARF with HFNC or NRS for 12 hours.	- IMV since the onset of hARF - treated with more than one therapy (e.g., HFNC/ NIV/CPAP) at the onset of hARF - awake PP - DNI order	NRS Group Interface: Not mentioned - PEEP 10-12cmH_2_O - PS 10-12 cmH_2_O	HFNC Group - Flow: 50-60 L/min	↔ IMV % ↔ Oxygenation	↑ 28-day Mortality in NRS Group
Colaianni-Alfonso et al. (35)(n = 112)	COVID Patients that failed in maintain RR < 30; SpO2 ⩾ 94% with FiO_2_ < 60% by HFNC	Pregnancy, hypercapnic patients and DNI patients	CPAP with face-mask (n = 57)	CPAP with Helmet (n = 55)	Helmet Group - ↓ IMV % - ↑ Oxygenation	Face-Mask Group: ↑ Mortality, LOS ↓ S / F, PEEP, Time to IMV
			CPAP: 10 – 14 cmH2O, FiO_2_ to SpO2 = 92-96%. 24h with continuous CPAP. After that, CPAP and HFNC were used alternatively			
Schmidt et al. (47) (n = 1491)	- No IMV on admission - > 16 years	IMV on the day of admission	COT, NIV, HFNC, or combined therapy Interface: bucconasal or facemask COT: 4-10 L/min NIV: PS 6–10 cmH2O, PEEP 6–8 cmH2O FiO2 50–80%. HFNC: Flow: 40–60 L/min and FiO_2_ was 60–90 %.	No control Group	↔ IMV % ↑ Mortality in ICU for NRS ↔ Hospital LOS ↑ Mortality at 28,60, 90^th^ day for NRS; ↔ for COT or HFNC	No described
Sivaloganathan et al. (48) (n = 101)	hARF	No described	NRS or IMV Group NRS only, NRS + IMV or IMV only	NRS Ceiling NRS as ceiling of treatment	↑ IMV in NRS or IMV Group ↓ Mortality in ICU in NRS or IMV Group ↑ Discharge in NRS or IMV Group	No described
**3) Randomized Controlled Trials (n = 3)**						
Grieco et al. (15) (n = 109)	- P/F ratio ≤ 200 mmHg - PaCO_2_ ≤ 45 mmHg, No history of chronic respiratory failure or moderate to severe cardiac insufficiency (NYHA > II or LVEF <50%),	- Acute exacerbation of chronic pulmonary disease; - Kidney failure - Previous treatment with NRS or HFNC at the time screening - Hemodynamic instability - Urgent IMV - DNI order - BMI > 40 - pH < 7,30 - Recent Thoracic or abdominal surgery - Cardiogenic oedema	NRS Group (48h continuous) Interface: Helmet - PEEP 10-12 cmH_2_O - PS 10-12 cmH_2_O - Esens: 10%-50% - FiO_2_ to SpO_2_ 92-98%	HFNC group (At least 48h - Flow: 60L/min initially. After 48h, FiO_2_ were titrate to maintain SpO_2_ 92-98%	↑ VFD at 28^th^ day in NRS Group	↓ IMV % in NRS Group; ↓ VFD at 60^th^ day in NRS Group; ↔- 28 and 60-day ICU Mortality ↔ 28 and 60-day Hospital Mortality ↔ ICU LOS ↔ Hospital LOS.
Perkins et al. (28) (n = 1273)	- hARF with SpO_2_ ≤ of 94% despite receiving COT with FiO_2_ ≥ 40%	- Immediate IMV - Known Pregnancy	CPAP Group Interface: Not mentioned - PEEP: 8.1-8.5 cmH_2_O	COT Group + HFNC Group Flow: 51.4-53.5 L/min	↓ IMV within 30 days in CPAP Group ↔ 30-day Mortality	↓ IMV % in CPAP Group ↔ Time in IMV ↔ ICU Mortality ↔ ICU LOS ↔ Hospital Mortality ↔ ICU Mortality
Arabi et al. (34) (N = 320)	- P/F ratio < 200 mmHg despite COT - COT > 10 L/min or above	- Immediate IMV - GCS < 12 - PaCO_2_ > 45mmHg - Pregnancy - Unstable Hemodynamic - Cardiopulmonary arrest - DNI Patients	NRS Interface: Helmet - PEEP 8-10 cmH_2_O - PS 10 cmH_2_O - FiO_2_ = 100% - Flow Rate > 50L/min - Rise time of 50ms - Esens of 50% - Maximum Pp = 30 cmH_2_O - PP - Light sedation if needed	Usual Respiratory Group: NRS with mask, HFNC or COT	↔ 28-day Mortality	↔ ICU Mortality ↔ Hospital Mortality ↔ ICU free days ↔ VFD ↔ IMV % ↔ Hospital LOS ↔ Time to IMV ↔ Kidney replacement ↔Vasopressin free days
Arabi et al. (37) (n = 317)	- P/F ratio < 200 mmHg despite COT - COT > 10 L/min or above - Suspected or confirmed COVID-19 pneumonia	Immediate IMV - GCS < 12 - PaCO_2_ > 45mmHg - Pregnancy - Unstable Hemodynamic - Cardiopulmonary arrest - DNI Patients	NRS Interface: Helmet - PEEP 8-10 cmH_2_O - PS 10 cmH_2_O - FiO_2_ = 100% - Flow Rate > 50L/min - Rise time of 50ms - Esens of 50% - Maximum Pp = 30 cmH_2_O - PP - Light sedation if needed	Usual Respiratory Group: NRS with mask, HFNC or COT	180-day mortality ↔ between groups QoF ↔ between groups	Absent

↑: increase, improvement; ↓: worsening, decrease; ↔: No difference; BMI: Body Mass Index; COT: Convention Oxygen Therapy; CPAP: Continuous Positive Airway Pressure; DNI: Do Not Intubate; Esens: Expiratory Sensibility; FiO_2_: Fraction of Inspired Oxygen; GCS: Glasgow Coma Scale; GGO: Ground Glass Opacities; hARF: hypoxemic acute respiratory failure; HFNC: High Flow Nasal Cannula; ICU: Intensive Care Unit; LOS: Length of Stay; LUS: Lung Ultrasound; LVEF: Left ventricular ejection fraction; IMV: Invasive Mechanical Ventilation; NRS: Non-invasive Respiratory Support; NYHA: New York Heart Association; P/F ratio: Partial Pressure of arterial oxygen (PaO_2_) to fraction of inspired oxygen (FiO_2_) ratio; PaCO_2_: Pressure of Arterial Carbon Dioxide; PBW: Predicted Body Weight; PEEP: Positive End Expiratory Pressure; PP: Prone Position; Pp: Plateau Pressure; PS: Pressure Support; RR: Respiratory Rate; S / F: SpO_2_ / FIO_2_ ratio; V_T_: Tidal Volume; VFD: Ventilator Free Days.

#### 3.6.6. Aerosol risk during NRS

At the beginning of the pandemic, there was great concern about the production of aerosols which would spread the SARS-CoV-2. The current recommendation stated that the patient should be allocated in a room with negative pressure, and undergo NIV therapy with a double branch circuit and antibacterial filter (54, 55). Whittley et al. (56) at the beginning of the pandemic, when comparing low and high flow oxygen therapy devices with NIV reported that high flow oxygen with NIV had the greatest particle dispersion capacity. After the reduction in the number of cases, the therapy became flexible to meet the demand. In the current scenario, NIV is no longer considered to be a large-scale aerosol-producing therapy (57, 58). Dell’Olio et al. (57) carried out a study that evaluated the production of aerosols in 4 regions around patients undergoing NIV with total face interface. The regions were 50, 80, 150 and 200 meters from the patients’ mouths. The results showed that only 21% of these regions were contaminated by SARS-CoV-2, indicating that NIV is a safe therapy.

Winslow et al. (58) compared COT, NRS, and HFNC in terms of virus shedding rates. Each group had 10 patients and the analysis was performed with the patient ventilating properly and with a cough stimulus. The authors concluded that NRS and HFNC have a low dispersion rate when compared to COT.

## 4. Prone position

Patients with COVID-19 who have an indication for MV need to be protectively ventilated to prevent VILI. For this, a plateau pressure (P_plat_) <30 cmH_2_O, driving pressure (ΔP) <15 cmH_2_O, and V_T_ between 6 and 8 mL/kg of PBW are recommended (59). However, within the pathophysiology of COVID-19, the patient may have poorly or non-ventilated lung areas, mainly in the basal and dorsal regions, in contrast to great aeration in the ventral regions, leading to hyperinflation (8). This is called pulmonary heterogeneity and may lead to low respiratory compliance. As a result, there is intrapulmonary shunt formation, mismatching the V/Q ratio (60). Thus, some patients may not respond to lung protective ventilation (LPV), requiring rescue maneuvers, such as PP (19, 20).

Recent studies have shown that COVID-19 has features of ARDS (61), allowing the Surviving Sepsis Campaign panel to recommend that the treatment of COVID-19 be similar to that of ARDS (12).

### 4.1. Effects of PP

PP is a non-pharmacologic strategy widely adopted in moderate/severe cases of ARDS with inadequate gas exchange (i.e., PaO_2_/FiO_2_ ratio < 150, with FiO_2_ > 60%) even with PEEP optimized within the concept of LPV. In ARDS, PP redistributes air volume from ventral to dorsal areas, promoting lung homogeneity (19, 20) because lung ventilation is dependent on gravity (20). PP also reduces regional lung stress/tension by displacing non-ventilated areas ventrally (20, 62, 63). Recruitment of the dorsal region of the lung is observed with subsequent increase in regional oxygenation and de-recruitment of the ventral region, leading to a decrease of the hyperinflated tissue (63, 64). In this case, a reduction of the dorsal shunt is observed, improving oxygenation (19, 20). Grasselli et al. (62) state that oxygenation can improve between 60 and 80%.

### 4.2. Ventilatory mechanics versus oxygenation

Final PaO_2_ is a weighted average of the PaO_2_ of blood flowing from different lung units. This means that the number of atelectatic units in the dependent lung regions is proportional to the severity of hypoxemia (63). In a supine position, with an angle of 0°, approximately 60% of the total lung mass is dependent. In COVID-19, perfusion irregularity promotes greater perfusion in these regions, leading to a decrease in the V/Q ratio (20, 62). During PP, however, only 40% are in the dependent position; i.e., fewer lung units are hyperperfused, resulting in better oxygenation (63). The consequence, in terms of ventilatory mechanics to the PP, is a decrease in total compliance of the chest wall, due to the functional stiffening of the anterior chest wall (63, 64). Thus, an improvement in lung compliance values and a more homogeneous V/Q distribution are expected (64). This also reduces VILI, resulting in improved parameters of ventilatory mechanics (49).

### 4.3. Patients eligible for prone position

The correct indication for PP is directly correlated with the duration of the disease and the patient’s clinical status. The L phenotype is characterized by moderate to severe hypoxemia, even with normal lung compliance (6). This phenotype is considered unresponsive to PP, and the observed improvement in oxygenation is due to the redistribution of blood flow from dorsal to ventral areas, without any alveolar recruitment, as seen in ARDS (42). This, PP in this phenotype does not bring great benefits, because this phenotype has no or little recruitment capacity. However, better aeration of dorsal regions is noted, reducing the chances of atelectrauma (64). Furthermore, COVID-19 is progressive, evolving to the H phenotype, which is more recruitable (6, 25). In this phenotype, there may be a worsening of lung compliance, without any relationship with the conduct. It is at this point that PP becomes more indicated. There is also an improvement in oxygenation, but at the expense of directing blood flow to dorsal regions with alveolar recruitment between patients (62). The ventilatory difficulty of the F phenotype contraindicates PP, because the benefits will be few. This is due to organizing pulmonary fibrosis (25, 27, 63). At this time, protective ventilation is prioritized (25).

The study by Fossali et al. (64) provides information relevant to the topic. The authors studied 21 patients with a mean age of 67 years. They performed chest computed tomography in a supine position and PP. Afterward, within the ICU, the authors performed electric impedance tomography (EIT) to verify distribution and ventilation and perfusion. All were protectively ventilated, without adjustments, in pressure regulated volume-controlled mode with PEEP maintained at 10 cmH_2_O. The authors described that there was no difference in the compliance of the respiratory system in both decubitus positions. The authors hypothesizes that in supine position, there may be alveolar units subject to cyclic openings and closings, which would be reduced in PP. In addition, another possible reason is that there was a decrease in lung elastance associated with increased chest wall rigidity. In addition, there was recruitment of dorsal regions, with perfusion improvement in these regions and de-recruitment of ventral regions. This allowed reduction in barotrauma and atelectrauma, reduction of areas with dead space, reducing the number of alveolar units with low V/Q, which improved V/Q matching. This dorsal de-recruitment is called spongelung (65) and is characterized by a reduction in dorsal pulmonary tension and ventral hyperdistention. The authors also point out that there was a reduction in the dead space/shunt ratio in PP and that this is also a marker of lung protection. However, the patients included in this study had been ill for an average of 8 days. Considering that COVID-19 is a progressive disease, it can be inferred that the patients were in phenotype transition to H and F, when the PP has few benefits.

The retrospective study by Langer et al. (66) divided 1,057 patients ventilating protectively into two groups (PP and supine position) with a mean age of 63 years. The average time to perform the first PP was 2 days. The authors observed that there was no difference in oxygenation and ventilatory mechanics between the groups. This can be explained by the high compliance at baseline. Therefore, the effect of PP may not work solely by recruitability, but through the redistribution of pulmonary blood flow.

Weiss et al. (19) studied 42 patients with a mean age of 59 years, but with significant obesity (body mass index (BMI) > 34 kg/m^2^), also under LPV. The researchers performed three PP sessions. In contrast to the article mentioned earlier, there was improvement in oxygenation after the first PP session, but a similar effect was not observed during the second and third PP sessions. This can be attributed to disease progression.

Recently, the COVID-19 Veneto ICU Network research group developed the PROVENT-C19 Registry, a large multicenter protocol specifically for patients with COVID-19 with the aim of describing the population that most benefits from PP (67). On admission, anthropometric data, data on comorbidities, and the type of ventilatory support used before EIT will be collected. The outcomes to be analyzed include differences in gas exchange and the PaO_2_/FiO_2_ ratio and ventilatory parameters before and after PP, prone duration, and ICU and hospital mortality. Considering the expected large population of this study, there will be an important improvement in clinical practice.

### 4.4. Duration of PP

The recommended duration of PP is at least 16 h (61, 68, 69). However, some studies have reported durations longer than 16 h of PP with different outcomes. The prospective study by Engerström et al. (70) evaluated 1,714 patients with a mean age of 64 years. The mean time between intubation and first PP session was 20.4 h. No association between early PP and survival was observed. Protti et al. (20) studied 15 patients with a mean age of 69 years and a mean BMI of 29 kg/m^2^. Patients were intubated within 2 days and were placed in PP within 3 days. There was a reduction in the volume of non-aerated gas and hyperventilated areas, indicating a lower possibility of VILI and an increase in respiratory compliance. An important point in this study is that the patients did not experience delayed intubation. This certainly has effects on the outcomes.

Encouraging results were also found in the study by Page et al. (60). The authors studied 52 obese patients (BMI >32 kg/m^2^) with a mean age of 62 years. They were randomized between conventional prone (16 h) and extended prone (24 h). There was no change in respiratory mechanics, but patients who remained prone longer had more ventilator-free days.

A longer time in the prone position was reported by Rezoagli et al. (71). The standard PP group lasted for 16 h, while extended PP consisted of 40 h. Although the extended PP group was younger than standard PP group, extended PP was feasible and was able to reduce the workload of health professionals. Taking into account the oppressive condition during pandemic, the reduction in workload is an important issue to consider. Furthermore, no benefits or harm in terms of gas exchange or respiratory mechanics were found when extended PP was compared to the standard PP group.

On returning to supine position, some patients may experience a decrease and loss of oxygenation gain (59, 72), further favoring extended PP. Recently, the retrospective study by Okin et al. (72) compared 267 patients with a mean age of 62 years who were subjected to 16 h and 24 h of PP in terms of mortality; 157 patients underwent extended PP (>24 h) and 110 underwent conventional PP (up to 16 h). The authors observed that mortality at 30 and 90 days was lower in the extended PP group. In addition, the study highlights that extended PP is safe, because it reduces the number of supine sessions that are associated with alveolar de-recruitment, increased atelectasis, and VILI, contributing to mortality. It also reduces the amount of neuromuscular blockers, reducing diaphragmatic dysfunction (72).

Thus, there is no limit on the number of PP sessions as long as they are recommended. For example, Walter et al. (73) reported that some patients underwent PP 22 times. The same study also suggests that PP should be interrupted when the FiO_2_ requirement is ≤60%, when the PaO_2_/FiO_2_ ratio is >150, and when the PEEP is ≤12 cmH_2_O.

### 4.5. Early or late PP?

Delaying PP is associated with higher mortality. The study by Mathews et al. (74) included 2,338 patients; 702 were placed in PP within 2 days of MV and the other 1,636 within 2 days of MV with a P/F ratio < 200. The authors observed that the early PP group had greater chance of developing shock and use of corticosteroids. However, the risk of death was lower. COVID-19 is a heterogeneous disease, therefore it is not possible to define a suitable time to implement PP. One suggestion is to use the same reasoning used to determine the need to transition from NRS to intubation: the worsening of compliance and the need for high FiO_2_ fractions to maintain adequate SpO_2_. In this case, it is possible to infer a change from the L to the H phenotype, which has a greater possibility of recruitment, benefiting from PP (25, 63).

With regard to the objectives of prone decubitus, associated articles describe the physiologic and ventilatory changes of the position. However, when analyzing the effects of PP, the increase in survival must be considered. Directing the therapeutic target only to improve oxygenation can be a scientific limitation. To guide the understanding of this topic, Table 2 contains a summary of the included studies and outcomes found.

**Table 2:** Selected studies on prone position.

Trial (sample size)	Population		Intervention		Outcome		
	Inclusion criteria	Exclusion criteria	Treatment	Control	Primary	Secondary	
Retrospective studies (*n* = 4)							
Camporota et al. (13) (*n* = 376)	Patients who received at least one session of PP for ≥12 h Intubated patients who met Berlin definition of ARDS	Not mentioned	COVID-19 ARDS (C-ARDS) group	ARDS group	↑ RM in COVID-19 group ↔ Oxygenation ↔ Mortality	Absent	
			LPV with V_T_ between 6 and 6.5 mL/kg PBW				
Weiss et al. (19) (*n* = 42)	- Intubated COVID-19 patients - Indication to PP	Pregnancy, Reintubation Previous PP at a referring hospital.	PP: ≥16 h LPV High PEEP low FiO_2_ tables If P/F in SP >150 mmHg, or ECMO or palliative care was needed, PP was terminated	Absent	↑ Oxygenation in 2nd PP session	↔ Discharge ↔ Hemodynamics ↔ RM	
Langer et al. (66) (*n* = 1,057)	Intubated patients who met Berlin definition of ARDS	Age < 18 years Noninvasive respiratory support Missing clinical data regarding the use of PP	PP group	SP group	SP group ↑ ICU survival ↑ Hospital survival ↑ ICU LOS ↑ Time on MV ↔ Hospital LOS	↑ Oxygenation in SP group ↔ RM, except for P_plat_ which was lower in the SP group ↔ PaCO_2_	
			LPV with V_T_ of 6.3–7.8 mL/kg PBW				
Hochberg et al. (68) (*n* = 512)	Intubated patients who met Berlin definition of ARDS Age > 18 years Indication for PP At least 72 h of IMV	Cardiac arrest Chronic IMV Tracheostomy as first airway IMV <48 h Contraindication for PP	COVID-19 ARDS	ARDS before pandemic	↓ Time to prolonged PP in COVID-19 group	In COVID-19 group ↑ Duration of PP ↑ PP sessions	
Prospective studies (*n* = 5)							
Protti et al. (20) (*n* = 15)	Diagnosis of ARDS Ongoing IMV PP prescribed by the attending physician within 3 days of IMV	Not described	(1) RM + CT in SP (2) PP + new CT + SP No adjustment of PEEP LPV with V_T_ between 6 and 7.1 mL/PBW	Absent	In PP group ↑ Oxygenation ↑ Lung aeration ↔ RM	Absent	
Le Terrier et al. (69)	P/*F* < 300 with PEEP >5 cmH_2_O	Not confirmed COVID-19 even with radiologic pattern	Early PP group	Non-early PP group	↔ Mortality at 60th day	↔ Mortality at 28th and 90th day In non-early group: ↓ VFD until 28th day ↓ ECMO ↓ NO ↓ Static compliance at 3rd day ↓ P/F at 3rd, 5th and 7th day	
Engerström et al. (70) (*n* = 1,714)	P/F ratio < 150 mmHg Patients receiving IMV within 24 h	Confirmed SARS-CoV2 with reason for admission other than COVID-19	Early PP group	Not early PP group	↔ Oxygenation ↔ 30-day mortality	↔ 90-day mortality	
Walter et al. (73) (*n* = 81)	ARDS COVID-19 intubated patients who had undergone at least one session of PP of >24 h duration Age ≥ 18 years P/F ratio < 150 mmHg	Missing data about PP session	PP ≥24 h iNO and ECMO were used if necessary LPV with V_T_ between 6 and 8 mL/kg PBW	Absent	↔ Pressure injuries between stage II and III	↑ Oxygenation ↑ RM	
			Ventilator mode is not described				
Mathews et al. (74)	P/*F* < 200 within 2 days of ICU admission	P/*F* > 200 ECMO on ICU day 1, cardiac arrest or severe arrhythmia Pronation before ICU admission Pregnancy	Early PP group		Late PP group	↓ Hospital deaths in early PP group	Absent
Randomized controlled trials (*n* = 1)							
Page et al. (60) (*n* = 52)	Patients intubated with: Age > 18 years Indication for PP	DNI patients Prisoner or pregnant IMV >48 h at the time of screening Any contraindication for PP	16 h of PP (traditional) + SP	24 h of PP (prolonged) + SP	↑ Time of PP session in prolonged PP	↔ Differences in RM ↔ Outcomes	
			LPV with V_T_ between 6 and 7 mL/kg PBW				

↑, increase, improvement; ↓, worsening, decrease; ↔, no difference; ARDS, acute respiratory distress syndrome; CT, computed tomography; DNI, do not intubate; ECMO, extracorporeal membrane oxygenation; FiO_2_, fraction of inspired oxygen; iNO, inhaled nitrous oxide; ICU, intensive care unit; IMV, invasive mechanical ventilation; LPV, lung protective ventilation; LOS, length of stay; NO, nitrous oxide; P/F ratio, partial pressure of arterial oxygen (PaO_2_) to fraction of inspired oxygen (FiO_2_); PaCO_2_, pressure of arterial carbon dioxide; PEEP, positive end expiratory pressure; PBW, predicted body weight; PP, prone position; P_plat_, plateau pressure; RM, respiratory mechanics; SP, supine position; V_T_, tidal volume.

## 5. Alveolar recruitment maneuvers and PEEP titration

At the beginning of the pandemic, there were doubts whether the pulmonary presentation of COVID-19 was similar to that of ARDS (51). A common factor is the difficulty in setting an ideal PEEP, although guidelines recommend the use of PEEP >10 cmH_2_O due to the large non-aerated area observed in COVID-19 (12). However, in some patients, oxygenation does not normalize, resulting in worse respiratory mechanics (i.e., ΔP >15 cmH_2_O; P_plat_ > 30 cmH_2_O), even during PP sessions.

### 5.1. Recruitability assessment

The use of PEEP tables, widely used for ARDS, is an easy alternative to titrate PEEP and sustain ARM (75–78). However, this strategy fails to optimize oxygenation; the PEEP response in patients with COVID-19 is highly heterogeneous due to the facts mentioned earlier (79–82). In this context, some studies chose the recruitment to inflation (R/I) ratio developed by Chen et al. (78) to assess the potential for recruitability in patients with ARDS. It ranges from 0 to 2. R/I < 0.5 indicates low potential for recruitability, increasing the risk of pulmonary overdistension without any benefit. R/I > 0.5 indicates high recruitability (79, 82, 83). After assessing recruitability, the choice of PEEP is based on ARM with decremental PEEP titration. Some studies have used only decremental PEEP titration (8, 78). Briefly, this strategy consists of gradually increasing the airway opening pressure up to 45 cmH_2_O and then performing PEEP titration (in steps of 2–3 cmH_2_O), maintaining the stability of hemodynamic and airway ΔP and allowing P_L_ to increase (80–83).

### 5.2. Effects of PEEP in oxygenation and perfusion

The issue of heterogeneity of oxygenation targets is a topic of discussion. Zerbib et al. (80) states that an SpO_2_ between 88 and 92% is satisfactory. Ball et al. (8) suggested that the best PEEP is the one in which PaO_2_ remains >60 mmHg. These two studies were less rigid about oxygenation, in contrast to previous studies dealing with ARM and PEEP titration. Randomized studies are needed to confirm whether these oxygenation targets are suitable for COVID-19.

### 5.3. Effects of PEEP with the L phenotype

For lungs with low recruitability (L-type phenotype; i.e., high static compliance), low levels of PEEP are sufficient to optimize PaO_2_ and reduce hyperdistended areas, P_plat_ and airway ΔP.

When high PEEP is applied to the L-type phenotype, it is expected to increase lung volume and reduce lung heterogeneity, at the cost of increased overinflated areas compared with low PEEP (8). Usually, airway pressure increases followed by impairment in respiratory system compliance. In the L phenotype, high PEEP values are not recommended, because this is a poorly recruitable phenotype (6, 8, 25). Increasing PEEP in this phenotype contributes to worsening lung compliance.

Pan et al. (76) studied 12 patients who were protectively ventilated; mean age was 59 years and the mean R/I ratio was 0.21, indicating low pulmonary recruitability. They showed that after applying high PEEP using the PEEP table (>15 cmH_2_O), P_plat_ remained high, with a low response in oxygenation. In addition, the authors reported that this patient profile may not respond to high PEEP in the supine position, but that recruitability seems to increase after PP. It can be inferred that this gain is due to displacement of poorly ventilated areas. This reinforces the fact that the PEEP table has partial applicability and seems to suggest that ARM should be performed together with PP.

### 5.4. Effects of PEEP in the H phenotype

When PEEP is applied incases with the H phenotype, the response is an improvement in lung compliance, with a reduction in P_plat_ and in poorly ventilated or non-ventilated areas, reducing intrapulmonary shunt (8, 25).

Protti et al. (20) studied 40 patients with early COVID-19 in the supine position and performed ARM plus decremental PEEP at three levels: 15, 10, and 5 cmH_2_O. With PEEP of 15 cmH_2_O, oxygenation improved in 36% of patients, but respiratory compliance improved in only 11%. There was also a reduction in non-ventilated areas and an increase in hyperventilated areas. Furthermore, two different responses were observed as PEEP increased. With an increase in PEEP from 5 to 10 cmH_2_O, recruitment was predominantly dorsal, reducing non-aerated tissue, with an improvement in the PaO_2_/FiO_2_ ratio and an increase in respiratory compliance. However, when PEEP was increased from 10 to 15 cmH_2_O, the recruitment obtained previously overlapped with the appearance of hyperventilated areas, predominantly ventral, and a decline in respiratory compliance. Furthermore, the improvement in oxygenation at high PEEP cannot be explained by recruitability, but rather by the improvement in left ventricular function, which decreases cardiac output (50).

–Ball et al. (8) studied a group of 42 recruitable and non-recruitable patients with a mean age of 63 years using LPV. The authors evaluated lung mechanics and oxygenation at two PEEP levels (8 and 16 cmH_2_O). The first group benefited from high PEEP by reducing the percentage of non-aerated lung units. However, only the non-recruitable group had a reduction in poorly aerated areas. Both groups showed improved oxygenation via increased hyperaerated areas, with consequent worsening of respiratory compliance. In practical terms, this led to an increase in ΔP, P_plat_, mechanical power, variables associated with VILI. The authors explained that the improvement in the P/F ratio should be interpreted as redistribution of the V′/Q′ ratio, prioritizing areas with low ventilation, and not as recruitment, even in so-called recruitable patients.

### 5.5. ARM and obese patients

Some of the studies discussed in this review analyzed obese patients, represented by BMI >30 kg/m^2^ (63, 64). Obese patients have a high recruitment potential and can tolerate high PEEP values, as long as the P_plat_ remains up to 30 cmH_2_O. Usually, studies have pointed out two main reasons for the need for high PEEP in this population: (1) decreased P_L_ (79); and (2) predominantly ventral ventilation with a tendency to dorsal alveolar collapse under low PEEP. This scenario can be prone to VILI due to low static compliance. After application of PEEP, the studies have highlighted decreased airway ΔP and dead space, with improvement in static lung compliance, P_L_, the PaO_2_/FiO_2_ ratio, and redistribution of pulmonary blood flow with subsequent reduction of intrapulmonary shunt (81).

Highly specialized centers have introduced EIT to expand understanding of the effect of PEEP levels (75, 81). EIT consists of placing a belt with electrodes between the fourth and fifth ribs to verify the ventilatory distribution (whether predominantly dorsal or ventral) in real time and macroscopically assess the effect of PEEP. The use of EIT during ARM and PEEP titration may guarantee the most adequate value for the patient, which may be two values below or above the values suggested by the PEEP table (75, 81). EIT shows the percentage of well-ventilated, poorly ventilated, collapsed, and hyperinflated areas; the latter two are of interest to the professional at the bedside to avoid VILI (49, 59).

### 5.6. The balance between oxygenation and ventilatory mechanics

Oxygenation is a therapeutic target, as is the assessment of ventilatory mechanics. Both need to be evaluated together and systematically. This review recommends that the search for the ideal P/F ratio, as well as optimal SpO_2_/PaO_2_ values, can lead to dangerous maneuvers of alveolar recruitment, exceeding protection limits, with the risk of P-SILI.

Beloncle et al. (10) studied 25 patients with COVID-19, 16 of whom were considered highly recruitable and 9 were considered poorly recruitable. Two PEEPs were applied: 5 and 15 cmH_2_O. At high PEEP, the recruitable group showed the same mean compliance for both PEEP levels. However, oxygenation in the recruitable group was higher than in the non-recruitable group. Ball et al. (11) studied 42 patients, 32 non-recruitable and 10 recruitable. The researchers applied two levels of PEEP (8 and 16 cmH_2_O). All patients then underwent computed tomography. They found that there was no percentage difference in recruitable areas despite the increase in PaO_2_. Therefore, it can be concluded that the compliance of the respiratory system can mitigate oxygenation. The articles of this topic were organized in Table 3, to direct the understanding of ARM.

**Table 3:** Selected studies on alveolar recruitment maneuvers.

Trial (sample size)	Population		Intervention				Outcome	
	Inclusion criteria	Exclusion criteria	Treatment	Control			Primary	Secondary
Retrospective studies (*n* = 6)								
Chiumello et al. (16) (*n* = 61)	Intubated patients who met the Berlin definition of ARDS	Barotrauma COPD Hemodynamic instability	PEEP: 5 cmH_2_O		PEEP: 15 cmH_2_O		↑ Oxygenation with PEEP of 15 cmH_2_O ↓ RM with PEEP of 15 cmH_2_O	Not mentioned
			VCV with LPV + RCM					
Sella et al. (75) (*n* = 15)	Intubated patients who met the Berlin definition of ARDS	Not mentioned	EIT-based PEEP group (group A)		PEEP/FiO_2_ tables group (group B)		↔ Oxygenation ↓ RM in group B	Not mentioned
			All patients were ventilated in VCV, with lung LPV					
Pan et al. (76) (*n* = 12)	Intubated patients who met the Berlin definition of ARDS	Not mentioned	VCV with 6 mL/kg PBW PEEP was set based on R/I ratio and P_plat_ 24-h session of PP and ECMO were discussed if necessary		Absent		↓ Mortality in PP ↑ Changes in RM in PP	Not mentioned
Van der Zee et al. (81) (*n* = 15)	Intubated patients who met the Berlin definition of moderate to severe ARDS	Not mentioned	Decremental PEEP trial + EIT + compare with PEEP/FiO_2_ table Minimum of PEEP of 24 cmH_2_O from baseline		Absent		↓ Oxygenation ↔ RM ↑ Lung aeration	Absent
Schulz et al. (83) (*n* = 27)	Intubated patients who met the Berlin definition of ARDS Age > 18 years Moderate or severe ARDS receiving IMV with ≥5 cmH_2_O PEEP RCM by increasing PEEP to +50% above the baseline	Pneumoperitoneum Pneumomediastinum undrained Pneumothorax or ongoing air leak Hemodynamic instability	PEEP_low_ responders (group A)	PEEP_low_ nonresponders (group B)	PEEP_high_ responder (group C)	PEEP_high_ nonresponders (group D)	↑ Oxygenation in group C	↔ Lung aeration
			VCV with LPV RR adjusted to permissive hypercapnia					
Bonny et al. (84) (*n* = 10)	Intubated patients who met the Berlin definition of ARDS	Not mentioned	PEEP: 16 cmH_2_O		PEEP: 8 cmH_2_O		↔ Oxygenation ↔ Hemodynamics ↓ RM with high PEEP	Absent
			VCV with V_T_ of 6–6.3 mL/kg PBW RR: 23–30 bpm FiO_2_: not mentioned					
Prospective studies (*n* = 6)								
Beloncle et al. (10) (*n* = 25)	Intubated patients who met the Berlin definition of ARDS R/I ratio ≥ 0.5	Age < 18 years Pneumothorax ECMO	Highly recruitable		Poorly recruitable		↔ Oxygenation ↔ RM ↔ Changes in hemodynamics	Absent
			Decremental PEEP: 15–10–5 cmH_2_O VCV with V_T_ of 6 mL/kg PBW					
Ball et al. (11) (*n* = 42)	Intubated patients who met the Berlin definition of ARDS	Not mentioned	Recruiters (low compliance)		Nonrecruiters (high compliance)		↔ Alveolar recruitment	↔ Oxygenation ↑ RM
			LPV CT scan at PEEP 8 cmH2O during expiratory breath-hold. Then, PEEP ↑ to 16 + New CT					
Rossi et al. (12) (*n* = 25)	Patients with confirmed COVID-19	Not mentioned	Supine: 5 cmH_2_O	Prone: 5 cmH_2_O	Supine: 35 cmH_2_O		↓ RM in supine: 35 cmH_2_O ↑ Lung aeration in supine: 35 cmH_2_O	Not mentioned
			(1) CT in SP and PP at 5 cmH_2_O of airway pressure (2) CT in SP at 35 cmH_2_O of airway pressure					
Somhorst et al. (77) (*n* = 75)	Age ≥ 16 years Intubated patients who met the Berlin definition of ARDS EIT availability	Contraindication to EIT belt Thoracic bandages Undrained pneumothorax Hemodynamic instability	PEEP ↓ to baseline	PEEP ↔ to baseline	PEEP ↑ to baseline		↔ Oxygenation ↑ RM in PEEP ↓ to baseline ↑ Lung aeration in PEEP ↑ to baseline	Not mentioned
			PCV with LPV From baseline PEEP: Use of EIT + decremental PEEP + small RCM					
Perier et al. (79) (*n* = 30)	Intubated patients who met the Berlin definition of ARDS	Contraindication to EIT (pacemaker, implantable defibrillator, skin lesion)	COVID-19 ARDS group		ARDS group		↔ Changes in RM ↔ Time in MV ↔ ICU LOS ↔ Death in ICU ↔ Need of vasopressors ↔ ECMO and tracheotomy %	Not mentioned
			V_T_ of 6 mL/kg PBW with initial PEEP: 18 cmH_2_O if P_plat_ remained <35 cmH_2_O Using EIT, PEEP was decreased by 3 cmH_2_O until reaching 6 cmH_2_O					
Zerbib et al. (80) (*n* = 30)	Intubated patients who met the Berlin definition of ARDS	P/F ratio > 150 mmHg Pneumothorax Pneumomediastinum Hemodynamic instability	Low recruitability (group A)		High recruitability (group B)		↔ Oxygenation ↑ RM in group B	Not mentioned
			VCV with LPV Performed RCM with maximum DP of 15 cmH_2_O					
Randomized controlled trials (*n* = 1)								
Protti et al. (82) (*n* = 40)	≤3 days of IMV	Pulmonary air leak Hemodynamic instability	PEEP: 5	PEEP: 10 cmH_2_O	PEEP: 15 cmH_2_O		↑ Oxygenation with PEEP 10 cmH_2_O ↓ RM with PEEP 10–15 cmH_2_O ↑ Lung aeration with PEEP 10–15 cmH_2_O	Absent
			All patients in SP RCM + CT at AWP of 45 and 5 cmH_2_O or CT at AWP of 15 and 5 cmH_2_O (group B) + PEEP trial (5, 10, and 15 cmH_2_O)					

↑, increase, improvement; ↓, worsening, decrease; ↔, no difference; ARDS, acute respiratory distress syndrome; AWP, airway pressure; COPD, chronic obstructive pulmonary disease; CT, computed tomography; ECMO, extracorporeal membrane oxygenation; EIT, electrical impedance tomography; FiO_2_, fraction of inspired oxygen; ICU, intensive care unit; IMV, invasive mechanical ventilation; LPV, lung protective ventilation; LOS, length of stay; P/F ratio, ratio of partial pressure of arterial oxygen (PaO_2_) to fraction of inspired oxygen (FiO_2_); PEEP, positive end expiratory pressure; PBW, predicted body weight; PCV, pressure-controlled ventilation; PP, prone position; P_plat_, plateau pressure; R/I ratio, recruitment to inflation ratio; RCM, recruitment maneuver; RM, respiratory mechanics (compliance, P_plat_, peak pressure); RR, respiratory rate; SP, supine position; VCV, volume-controlled ventilation; V_T_, tidal volume.

## 6. Extracorporeal membrane oxygenation

The administration of low V_T_ in severely collapsed lungs results in increases in CO_2_ levels (i.e., >45 mmHg) leading to the development of respiratory acidosis and extremely severe hypoxemia (84). Patients with extensive alveolar consolidations are likely to be refractory to the PP and ARM maneuver with decremental PEEP (84, 85). Analysis of lung mechanics demonstrates P_plat_ and ΔP above protective limits (30 and 15 cmH_2_O, respectively), and pH less than 7.35 (85, 86). This clinical picture could benefit from ECMO.

ECMO is a potentially life-saving strategy recommended in patients who are extremely hypoxemic and acidotic, with the aim of clearing CO_2_ levels and allowing the lungs to reduce activity, allowing the ECMO to perform gas exchange. Due to its high complexity, use of ECMO is recommended only in specialized centers and by dedicated staff (87). The studies in this review were based on the ELSO (Extracorporeal Life Support Organization) and EOLIA (ECMO to Rescue Lung Injury in Severe ARDS) definitions to define patients eligible or not for therapy. Among so many recommendations, we highlight: (1) PaO_2_/FiO_2_ ratio < 50 mmHg over 3 h; (2) PaO_2_/FiO_2_ ratio < 80 mmHg over 6 h; (3) arterial blood gas pH <7.25 and PaCO_2_ > 60 mmHg over 6 h (Figure 2).

There are different ventilatory strategies during ECMO. The randomized clinical trial by McNamee et al. (87) studied 412 patients with severe hypoxemia and < 48 h of intubation and randomized them into two groups: (1) ECMO + LPV and (2) LPV only. The first group showed a reduction in P_plat_ and ΔP and more ventilator-free days, indicating improved lung protection compared with the second group. No difference in mortality was found.

### 6.1. Time to start ECMO

Considering the inclusion criteria for ECMO, it is pertinent to consider its rapid start after detection of the disorder. There is no consensus in the literature about when to start therapy, because this depends on the availability of equipment and trained staff (85). Even so, the prospective study Mustafa et al. (88) studied 160 patients with a mean age of 49 years. The researchers divided them into two groups: (1) ECMO + LPV; (2) Only MVA. The first group progressed to ECMO within 3.8 days. ECMO + LPV was associated with 68% survival, whereas LPV only was associated with 26% survival. Karagiannidis et al. (89) stated that ECMO should start within 3 days because it is associated with longer patient survival. The multicenter study by Lorusso et al. (90) analyzed 1,215 patients ventilated with a V_T_ < 3 mL/PBW and concluded that age > 60 years and a time longer than 4 days between the start of MV and the start of ECMO was associated with higher mortality.

Recently, Hajage et al. (91) studied 2,858 patients; 269 (mean age, 53 years) received ECMO within 14 days of hospitalization. Patients were intubated within 1 day of hospitalization, the average time to start ECMO was 6 days, and 89 and 97% of patients received PP and neuromuscular blockers, respectively, before ECMO. All patients were ventilated ultraprotectively, i.e., V_T_ < 4 mL/kg. It was observed that eligible patients had poor ventilatory mechanics, with a mean ΔP of 18 and a mean P_plat_ of 30 cmH_2_O. The results showed that there was a significant improvement in ΔP to 12 cmH_2_O and P_plat_ to 18 cmH_2_O within 48 h of ECMO. These results are encouraging and reinforce the recommendations for successful ECMO: young age, few days of MV, and few comorbidities.

ECMO is a high-cost strategy that requires highly trained staff (85, 87), which may limit its widespread application and/or late start, when the patient may not benefit from the therapy.

### 6.2. Eligible patients

Preliminary prospective results from Kon et al. (92) highlighted important issues. The authors studied 27 obese patients with a mean age of 40 years. The authors chose to include only functional independent patients without comorbidities in the study. Before being eligible for ECMO, patients were LPV with a mean PEEP of 14 cmH_2_O and FiO_2_ > 90%. The primary endpoint of the study was survival during hospitalization and lung recovery (defined by the authors as ECMO weaning). They reported that 11 patients fully recovered on ECMO, and 13 were still on ECMO. The recovered group was successfully decannulated. All patients were tracheostomized with a median time of 24 h, allowing for lower rates of sedation and neuromuscular blockers, in addition to reducing the possibility of nosocomial infections, in contrast to other studies that reported at least 2 days from intubation to ECMO. All patients were ventilated in volume-controlled ventilation mode with 5 mL/kg PBW, with a mean PEEP of 10 cmH_2_O. The patients had median low compliance (22 mL/cmH_2_O) and ΔP ranging from 14 to 18 cmH_2_O. In addition, patients had a mean PaCO_2_ of 80 mmHg, pH <7.25, and mean serum lactate levels of 2.45 mmol/L. The primary endpoint of the study was 90-day mortality. All these factors were associated with mortality, which was 38.8%. Therefore, the main success factor for ECMO is young age, indicating the need for correct selection of patients (89).

The study by Schmidt et al. (21) included 83 patients, 30 of whom died. Forty-eight patients survived and were discharged from the ICU. The average age was 48 years. Interestingly, the surviving group had higher mean d-dimer values than the group who died. Moreover, 88% of the patients were ventilated in airway pressure release ventilation (APRV) mode, known to ensure alveolar stability and allow for greater pressurization with reduced occurrence of VILI (93). This ventilation mode was not used in almost all of the articles cited that opted for volume-controlled ventilation or pressure-controlled ventilation. Mortality was 31%. There was no information about the association of APRV and the effects of ECMO. But given the purpose of the APRV, it is possible to infer that the association would behave as double lung protection. Other studies associating ECMO and APRV are needed to confirm the positive relationship between them.

### 6.3. PP on ECMO

Although there are few studies reporting the use of PP in ECMO, recent evidence points to a good response from the combined therapies. Garcia et al. (94) studied 25 patients with COVID-19 that required V/V ECMO. 14 were placed on PP at least once for 16 h on average. All of them were protectively ventilated. In terms of lung mechanics, there were no statistical differences between PP and non-PP patients. However, there was an improvement in oxygenation in the PP group. Massart et al. (95) evaluated 517 patients with a mean age of 55 years on ECMO; 364 were prone during therapy and 153 were not prone. All were protectively ventilated. Lower mortality rates were observed in the PP group. There was no statistical difference between lung compliance and gas exchange values. As with PP, the outcome that should guide clinical practice is mortality. Only randomized studies will be able to confirm if the improvement in oxygenation is due to ECMO or PP or to joint therapy.

### 6.4. Side effects of ECMO

Despite its beneficial effects, the articles cited here highlight that ECMO presents a high risk of bleeding requiring anticoagulation, and many patients progress to hemodialysis (53, 85–88, 94–98) These facts, added to the fibrotic evolution of COVID, increase the chances of mortality and therapeutic failure with ECMO. However, these factors may have less impact on young patients and/or those with few or no comorbidities. The positive and negative outcomes of the ECMO studies are shown in Table 4.

**Table 4:** Selected ECMO studies.

Trial (sample size)	Population		Intervention		Outcome	
	Inclusion criteria	Exclusion criteria	Treatment	Control	Primary	Secondary
Retrospective studies (*n* = 1)						
Herrmann et al. (97) (*n* = 673)	Age ≤ 70 years IMV <8 days before ECMO BMI ≤45 kg/m^2^ Absence of malignancies. no history of myocardial infarction Congestive heart failure	Age > 70 years Chronic pulmonary disease Kidney disease	In hospital survivors (group A)	In hospital nonsurvivors (group B)	↓ Mortality at 6th month in group A	In group A: ↓ Duration of ECMO ↑ ICU LOS ↑ Hospital LOS ↓ In hospital complications In group B: ↑ No. of PP before ECMO ↔ RM ↔ Time to ECMO
			V/V ECMO PP was applied if necessary LPV with TV ≤ 6-8 mL/PBW			
Prospective studies (*n* = 6)						
Schmidt et al. (22) (*n* = 159)	EOLIA/ELSO criteria	>70 years Severe comorbidities Cardiac arrest Multiorgan failure or SAPS II >90 Irreversible neurologic injury IMV for >10 days	Patients alive V/V or V/A ECMO	Dead patients V/V or V/A ECMO	↑ RM in dead patients group ↔ Oxygenation	Absent
			Blood flow: 4.0–5.5 L/min Sweep gas: 3–7 L/min FDO_2_ = 100% LPV with V_T_ between 1.4 and 4.2 mL/kg PBW			
Mustafa et al. (88) (*n* = 160)	EOLIA/ELSO criteria	Patients not mechanically ventilated Cardiac arrest Lactate ≥14 mmol/L or pH ≤6.9 Multi-system organ failure Neurologic injury Recent hemorrhagic stroke Refuse to receive blood transfusion DNI patients Chronic organ failure Tumors Severe chronic disease requiring oxygen therapy	V/V ECMO	MVA patients*	↑ Survival ↑ VFD in MVA patients	↓ Time in IMV in MVA patients ↑ % mortality in MVA patients ↔ Oxygenation
Lebreton et al. (96) (*n* = 302)	EOLIA/ELSO criteria	Age > 70 years Serious comorbidities Multiple organ failure IMV for >10 days Cardiac arrest SAPS >90 Irreversible neurologic injury	Alive patients	Dead patients	Dead patients: ↓ ICU LOS ↑ ICU complications ↓Time in ECMO ↓ ECMO complications ↑ Mortality at 90th day after initiation of ECMO ↑ Organ dysfunction ↔ RM and PP sessions	Not mentioned
			V/V, V/A, VV/A ECMO sweep gas: 4–8 L/min Blood flow: 4.3–5.5 L/min LPV with V_T_ ≤ 4.9–6.2 mL/kg PBW			
Yang et al. (85) (*n* = 21)	EOLIA/ELSO criteria No response to PP RR >35 bpm P_plat_ > 30 cmH_2_O	Not mentioned	V/V ECMO + LPV	LPV only	↔ Mortality	↔ Complications associated with ECMO
			LPV in PCV with V_T_ of 4 mL/kg PBW			
Garcia et al. (94) (*n* = 60)	IMV + P/F ratio < 80 mmHg with FiO_2_ and FDO_2_ at 100% Extensive lung consolidation on CT	Not mentioned	V/V ECMO + PP group	V/V ECMO + SP group	SP group ↑ ECMO weaning ↓ Duration of ECMO ↓ Mortality at 28th day ↑ ICU discharge ↔ RM	Not mentioned
			Ultra LPV with V_T_ of 1.8–2.7 mL/kg PBW			
Whebell et al. (98) (*n* = 243)	EOLIA/ELSO criteria No response to PP in ≥6 h No response to LPV	Non-COVID-19 diagnosis	V/V ECMO	Conventional care	↓ Hospital mortality in ECMO group ↔ RM	Not mentioned
			LPV with V^T^ ≤ 6–8 mL/kg PBW			
Randomized controlled trials (*n* = 1)						
McNamee et al. (87) (*n* = 412)	hARF + IMV with PEEP ≥5 cmH_2_O 48 h with P/F ratio ≤ 150 mmHg	IMV >7 days Contraindication to heparin Untreated pulmonary embolism Pleural effusion or pneumothorax, or hARF fully explained by left ventricular failure or fluid overload	LPV + V/V ECMO Sweep gas Flow: 10 L/min LPV with V_T_ of ≤3 mL/kg PBW	Only LPV	↔ Mortality at 90th day	↑ VFD at 28th day in LPV ↔ Time in IMV ↔ Need for ECMO on 7th day ↔ Mortality at 28th day ↑ Adverse event in ECMO group

↑, increase, improvement; ↓, worsening, decrease; ↔, no difference; ARDS, acute respiratory distress syndrome; BMI, body mass index; COT, conventional oxygen therapy; CPR, cardiopulmonary resuscitation; CT, computed tomography; DNI, do not intubate; ECMO, extracorporeal membrane oxygenation; EOLIA, ECMO to Rescue Lung Injury in Severe ARDS; ELSO, Extracorporeal Life Support Organization; FDO_2_, fraction of oxygen in the sweep gas flow; FiO_2_, fraction of inspired oxygen; hARF, hypoxemic acute respiratory failure; ICU, intensive care unit; IMV, invasive mechanical ventilation; LPV, lung protective ventilation; LOS, length of stay; P/F ratio, ratio of partial pressure of arterial oxygen (PaO_2_) to fraction of inspired oxygen (FiO_2_); PaCO_2_, pressure of arterial carbon dioxide; PEEP, positive end expiratory pressure; PBW, predicted body weight; PCV, pressure-controlled ventilation; PP, prone position; P_plat_, plateau pressure; SP, supine position; RM, respiratory mechanics; SAPS, Simplified Acute Physiology Score; SpO_2_, oxygen saturation; TCT, tracheotomy; VCV, volume-controlled ventilation; V/A, venoarterial; V_T_, tidal volume; V/V, venovenous; VV/A, venovenoarterial. *MVA, maximized ventilator adjustments: FiO_2_ ≥ 80%, PEEP ≥ 10 cmH_2_O, and V_T_ 6 mL/kg PBW, keeping P_plat_ ≤ 32 cmH_2_O.

### 6.5. ECMO in non COVID-ARDS versus COVID-ARDS patients

Some studies compared ECMO in non-COVID-ARDS patients and COVID-ARDS patients. Although similar results were gathered about oxygenation (99, 100), the treatment time and complications were different. Chandel et al. (99) analyzed 9,271 patients who required ECMO between 2017 and 2021. Authors showed that COVID patients remained longer on ECMO when compared to non-COVID patients (19.6 days versus 10 days). Additionally, COVID patients had higher rates of developing kidney failure, requiring hemodialysis. Furthermore, COVID patients remained longer on mechanical ventilation before starting ECMO. This condition may lead to increased diaphragmatic dysfunction and mortality in COVID compared with non-COVID group. Other complications also observed in the COVID group included pneumothorax and intracranial hypertension. This can be explained by the high inflammatory cascade due to COVID.

Similar results were found in the retrospective study by Dave et al. (100). The authors studied 89 patients who used V/V ECMO, divided in two groups: 35 COVID patients and 54 non-COVID patients. COVID patients had higher in-hospital mortality rates (49% versus 24%), longer ECMO and mechanical ventilation time before ECMO (654 h versus 394 h; 3 versus 1 day, respectively) than non-COVID patients.

## Conclusion

This narrative review with a literature search strategy concludes that NRS, PP, ARM with decremental PEEP, and ECMO are therapeutic strategies that should only be applied in strictly selected patients. Noninvasive ventilatory support should be the therapy of choice with the aim of improving hypoxemia and ventilatory work. If no improvement is seen, orotracheal intubation should be instituted with a protective strategy. In cases of inefficient gas exchange, i.e., P/F ratio < 150, PP and ARMs can be performed provided that the patient has recruitability potential. ECMO should only be instituted in patients who, on MV for a short time, have inefficient gas exchange. However, ECMO needs a trained team, and its use is recommended only in highly specialized centers.

## Author contributions

LR, CR, DB, PP, PR, and PS wrote the manuscript and revised the final version. All authors read and approved the final version of the manuscript.

## Funding

The Brazilian Council for Scientific and Technological Development (CNPq) funded research projects and scholarships for students, Rio de Janeiro State Research Foundation (FAPERJ) funded research projects and scholarships for students, Coordination for the Improvement of Higher Education Personnel (CAPES) funded publication costs and scholarships for students, and the National Institute of Science and Technology for Regenerative Medicine/CNPq funded research projects.

## Conflict of interest

The authors declare that the research was conducted in the absence of any commercial or financial relationships that could be construed as a potential conflict of interest.

## Publisher’s note

All claims expressed in this article are solely those of the authors and do not necessarily represent those of their affiliated organizations, or those of the publisher, the editors and the reviewers. Any product that may be evaluated in this article, or claim that may be made by its manufacturer, is not guaranteed or endorsed by the publisher.
